# Kaempferitrin's Dual Assault: Inducing Apoptosis and Ferroptosis in Diffuse Large B‐Cell Lymphoma via NF‐κB Inactivation

**DOI:** 10.1002/kjm2.70110

**Published:** 2025-10-01

**Authors:** Han‐Shuo Zhang, Xiao‐Dan Zhou, Jin Chen, Ling Wang

**Affiliations:** ^1^ Department of Hematology Nantong First People's Hospital and Affiliated Hospital 2 of Nantong University Chongchuan District, Nantong Jiangsu China

**Keywords:** apoptosis, diffuse large B cell lymphoma, ferroptosis, kaempferitrin, NF‐κB

## Abstract

Diffuse large B‐cell lymphoma (DLBCL) is the most common non‐Hodgkin lymphoma and is an aggressive and highly heterogeneous tumor. Kaempferitrin (KPF) is a natural flavonoid glycoside that exerts a protective role in multiple human tumors. However, the impact of KPF on DLBCL remains unclear. In this study, we discovered that 240 μM KPF had a toxic effect on GM12878 cells. KPF inhibited DLBCL cell proliferation while promoting apoptosis in these cells. Additionally, KPF induced ferroptosis in DLBCL cells by elevating intracellular Fe^2+^ levels and reactive oxygen species (ROS) levels, alongside reducing the protein levels of GPX4 and SLC7A11. Moreover, KPF suppressed the activation of NF‐κB in DLBCL cells. Building upon this finding, we further validated that KPF reduced DLBCL cell malignant growth through the inhibition of NF‐κB activation. Meanwhile, animal studies further suggested that KPF inhibited DLBCL proliferation in vivo, mainly through reduced subcutaneous tumor volume, tumor weight, and increased apoptosis levels in mice. Furthermore, KPF suppressed the disorder of DLBCL cancer tissue arrangement and decreased p‐NF‐κB and p‐IKB‐α protein levels in DLBCL subcutaneous tumor tissues. In summary, our findings suggested that KPF enhanced apoptosis and ferroptosis in DLBCL cells via the deactivation of the NF‐κB signaling pathway.

## 1 Introduction

Diffuse large B‐cell lymphoma (DLBCL) represents the most common subtype of non‐Hodgkin lymphoma and exhibits high aggressiveness along with considerable heterogeneity [1]. According to classifications established by the World Health Organization, DLBCL is classified following cell of origin and is divided into germinal center B‐cell, or activated B‐cell subtype [2]. Recent treatment strategies for patients diagnosed with DLBCL predominantly involve combinations of rituximab with chemotherapy agents such as cyclophosphamide, doxorubicin, vincristine, and prednisone [3]. Unfortunately, up to 40% of DLBCL patients exhibit refractory disease or short‐term relapse, which is fatal for most patients [4]. Consequently, there is an urgent need for novel therapeutic agents aimed at improving survival in DLBCL patients.

Flavonoids are among the most prevalent natural compounds found in plants, including legumes, umbelliferae, salicyliaceae, and various plant tissues [5]. Flavonoids possess multiple biological activities, consisting of antioxidant, and anti‐inflammatory effects [6]. Flavonoids have also been shown to inhibit tumor growth and induce programmed cell death, suggesting that their anti‐tumor effects may provide novel insights into the development of therapeutic agents [7]. Kaempferol (KPF, kaempferol 3,7‐dirhamnoside) is a potent flavonoid compound that is isolated from *Bauhinia forficata* leaves [8]. As has been reported, KPF possesses antioxidant, anti‐inflammatory, anti‐apoptotic, pro‐apoptotic, cardioprotective, and anticancer activities [9]. In ongoing research, KPF has demonstrated an ability to alleviate various types of human malignant tumors. For example, KPF enhances apoptosis in liver cancer cells via the p21/Bcl‐2/Caspase‐3 signaling pathway, thereby providing a theoretical basis for its clinical application in liver cancer therapy [10]. Additionally, KPF induces G1 phase cell cycle arrest and apoptosis through a Caspase‐dependent intrinsic pathway, indicating its potential anti‐tumor effects in cervical cancer [11]. However, the regulatory mechanisms of KPF in DLBCL remain largely unknown.

In this study, our data preliminarily demonstrated that KPF repressed DLBCL cell proliferation and enhanced cell apoptosis. On this basis, this research further explored the possible role and mechanism of KPF in vivo and in vitro models of DLBCL, aiming to provide a theoretical basis for the clinical application of DLBCL.

## 2 Materials and Methods

### 2.1 Cell Culture

Normal human B lymphocytes (GM12878) were obtained from Sunncell (Wuhan, China). DLBCL cells (OCI‐LY1) were provided by Fenghbio (Hunan, China). DLBCL cells (U2932) were purchased from Sunncell. All cells were cultured in RPMI‐1640 (Procell, Wuhan, China) containing 10% FBS (Procell). The cells were maintained at 37°C in a humidified atmosphere containing 5% CO_2_.

### 2.2 Cell Treatment

To evaluate the toxic effect of KPF (#BBP00574, 578.52, C_27_H_30_O_14,_ 98.5%, Biobiopha, Yunnan, China) on GM12878 cells, GM12878 cells were treated with KPF (0, 3.75, 7.5, 15, 30, 60, 120 μM) for 1 day [11].

To explore the effect of KPF on DLBCL cell proliferation, DLBCL cells (OCI‐LY1 and U2932) were exposed to KPF (0, 3.75, 7.5, 15, 30, 60 μM) for 1 day [12]. Additionally, DLBCL cells were exposed to 30 μM KPF for 1 day. Then, the medium containing KPF was discarded. The cells were further exposed to either 5 mmol/L erastin (#HY‐15763, 547.04, C_30_H_31_ClN_4_O_4,_ 99.62%, MedChemExpress, Shanghai, China) or 1 mmol/L ferrostatin‐1 (#HY‐100579, 262.35, C_15_H_22_N_2_O_2,_ 99.71%, MedChemExpress) for 1 day [13, 14]. DLBCL cells were exposed to 10 μM benzyloxycarbonyl‐Val‐Ala‐Asp(OMe)‐fluoromethylketone (Z‐VAD‐FMK, the apoptosis inhibitor) for 1 day [15]. Followed by DLBCL cells (OCI‐LY1 and U2932) were treated with KPF (30 μM) for 1 day. Then, the medium containing KPF was discarded. The cells were further exposed to the medium containing 5 ng/mL TNF‐α for 3 days [16, 17].

To explore the effect of ferrostatin‐1 on KPF‐induced apoptosis of DLBCL cells, DLBCL cells were treated with 1 mmol/L ferrostatin‐1, 30 μM KPF, or 1 mmol/L ferrostatin‐1 combined with 30 μM KPF for 1 day. Then, cell apoptosis was tested using flow cytometry.

To further illustrate the KPF effect on the NF‐κB axis, U2932 cells were exposed to 30 μM KPF for 1 day. Then, the medium containing KPF was discarded. U2932 cells were further treated with the medium containing 100 ng/mL RANKL (activator for NF‐κB, #HY‐P73388, MedChemExpress) for 1 day [18]. DLBCL cells were treated with 30 μM KPF, 0.5 mmol/L *N*‐acetylcysteine (NAC, a potent scavenger of ROS, #HY‐B0215, MedChemExpress), or 30 μM KPF combined with 0.5 mmol/L NAC for 1 day [19].

### 2.3 Cell Counting Kit‐8 (CCK‐8) Assay

DLBCL cells (5 × 10^3^) were seeded into 96‐well plates and cultured in incubators for 1 day. Subsequently, 10 μL of CCK‐8 solution (MedChemExpress) was added to each well, and the cells were further incubated for 1.5 h. Absorbance (OD) at 450 nm was measured with a microplate reader (Thermo Fisher Scientific, Shanghai, China). In addition, a maximum of 50% inhibitory concentration (IC_50_) was calculated using the GraphPad Prism program (California, USA).

### 2.4 Flow Cytometry

DLBCL cell apoptosis was evaluated using the Apoptosis Detection Kit (#KGA1030, KeyGen Bio Tech, Nanjing, China). Harvested DLBCL cells were washed with pre‐cooled PBS (Thermo Fisher Scientific) and centrifuged (4°C, 300 × g) for 3–5 min. The cells were then resuspended in 1 × binding buffer and incubated with 5 μL of PE‐conjugated annexin V and 10 μL of propidium iodide (PI) staining solution for 15 min in the dark. Following incubation, 1 × binding buffer (400 μL) was added, and apoptosis was examined via flow cytometry (#Cytoflex, Beckman). FlowJo 10 software (Tree Star, California, USA) was applied to analyze data.

### 2.5 Detection of Fe^2+^ and Reactive Oxygen Species (ROS) Levels

To quantify Fe^2+^ levels in DLBCL cells and tissues, a Fe^2+^ assay kit (Abcam, Shanghai, China) was used in accordance with the manufacturer's instructions.

ROS production in DLBCL cells was evaluated using reactive oxygen species Assay Kits (Yeasen, Shanghai, China). Briefly, DLBCL cells were prepared as a single‐cell suspension and incubated in the dark with the H_2_DCFH‐DA fluorescent probe for 25 min. Afterward, H_2_DCFH‐DA that did not enter cells was removed. Eventually, DLBCL cells were observed under a fluorescence microscope (Zeiss, Shanghai, China).

### 2.6 Western Blot

Total protein was extracted from DLBCL cells using RIPA lysis buffer (Solarbio, Beijing, China). After quantifying protein concentration, protein was isolated on a 10% SDS‐PAGE (Solarbio) and then transferred to a polyvinylidene fluoride (PVDF) membrane (Thermo Fisher Scientific). The membranes were blocked with 5% non‐fat milk and incubated with primary antibodies against Glutathione Peroxidase 4 (GPX4, 1:2000, Abcam, ab125066), IKK α/β (1:1000, Abcam, ab32041), p‐IKK α/β (1:500, Abcam, ab38515), p‐NF‐κB (1:1000, Abcam, ab76302), NF‐κB (0.5 μg/mL, Abcam, ab16502), p‐IKBα (1:1000, Abcam, ab92700), IKBα (1:3000, Abcam, ab32518), and GAPDH (1:2000, Abcam, ab8245) incubated overnight at 4°C. Then, the membrane and secondary antibody (1:3000, Abcam) were soaked at room temperature for 1 h. Protein bands were evaluated using an enhanced chemiluminescence reagent (Beyotime). Optical densitometry analysis was executed using Image J (NIH, Bethesda, Maryland, USA).

### 2.7 Tumor Xenograft Model

All animal procedures were conducted in accordance with guidelines established by our Hospital's Ethics Committee. A total of 12 female NOD/SCID mice (6 weeks old, 20 ± 2 g) were obtained from Cyagen (Suzhou, China) and maintained under specific pathogen‐free conditions.

For in vivo research of KPF, U2932 cells (1 × 10^7^) were subcutaneously injected into the mice [20]. When tumor volume reached approximately 100 mm^3^, KPF (20 mg/kg) was intraperitoneally injected into DSCLC mice [21]. Weight and tumor volume were tested weekly for 5 weeks. The tumor volume formula was *V* = (*a* × *b* ^2^/2), where a was the largest size and b was the vertical diameter. After 5 weeks, all tumor tissue was gathered for follow‐up studies.

### 2.8 Hematoxylin–Eosin (HE) Staining

DLBCL tissues were fixed in 10% formaldehyde solution (Nanjing Reagent, Nanjing, China) for 2 days. Following fixation, DLBCL tissues were embedded in paraffin and sliced to 5 μm thick. Next, slices were stained with hematoxylin staining solution (Shzysw, Shanghai, China) for 3 min, and were then washed under running water. Slices were exposed to an eosin staining solution (Shzysw) for 2 min. Morphological changes were examined using a light microscope (Zeiss).

### 2.9 TUENL Assay

Paraffin‐embedded tissue sections (5 μm thick) were treated with DNase‐free protease K (20 μg/mL, Thermo Fisher Scientific) at room temperature for 20 min. Afterward, slices were treated with TUNEL solution (Thermo Fisher Scientific) for 1 h without light. The nucleus was dyed with Hoechst (Thermo Fisher Scientific). Images were observed under a fluorescence microscope (Zeiss).

### 2.10 Immunohistochemistry (IHC)

Following fixation and paraffin embedding, xenograft‐derived DSCLC tissues were sectioned into 4 μm slices. Then, slices were incubated with anti‐GPX4 (1:200, Abcam, ab125066), followed by incubation with HRP‐conjugated secondary antibody (1:2000, Abcam). The photographed images were gathered using an optical microscope (Zeiss).

### 2.11 Statistical Analysis

SPSS 20.0 (SPSS, Chicago, IL, USA) was applied for all statistical analyses, and the results were expressed as mean ± SD. Statistical comparisons were made by Student's *t*‐test between two different groups and one‐way analysis of variance (ANOVA) followed by Tukey's post hoc test among three or more groups. *p*‐values of less than 0.05 were considered statistically significant.

## 3 Results

### 3.1 KPF Suppresses DLBCL Cell Proliferation and Induces Apoptosis

**FIGURE 1:**
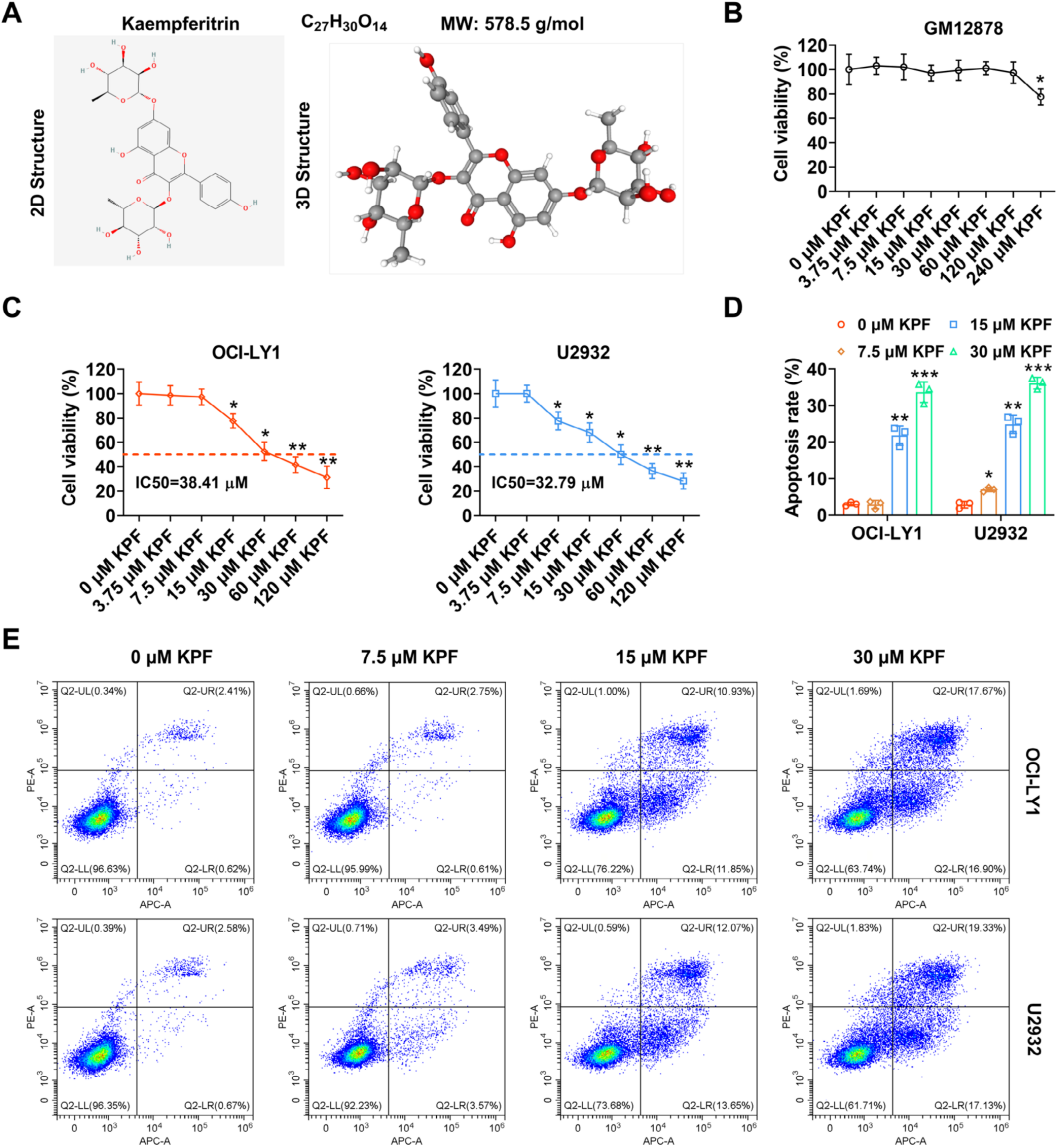
Kaempferitrin weakens diffuse large B‐cell lymphoma cell proliferation and enhances apoptosis. (A) The structural formula and molecular weight of Kaempferitrin (KPF, molecular weight: 578.52; Formula: C_27_H_30_O_14_). (B) Normal human B lymphocytes (GM12878) cells were treated with KPF (0, 3.75, 7.5, 15, 30, 60, 120 μM) for 1 day. Contrast of GM12878 cell proliferation via Cell Counting Kit‐8 (CCK‐8) analysis. (C) DLBCL cells (OCI‐LY1 and U2932) were exposed to KPF (0, 3.75, 7.5, 15, 30, 60, 120 μM) for 1 day. DLBCL cell proliferation was examined with the CCK‐8 assay. (D, E) OCI‐LY1 and U2932 were treated with KPF (0, 7.5, 15, 30 μM) for 1 day. DLBCL cell apoptosis was determined via flow cytometry. *p < 0.05, **p < 0.01, ***p < 0.001 versus 0 μM KPF. KPF: Kaempferitrin.

To evaluate the cytotoxic effects of KPF on normal human B lymphocytes (GM12878), we first determined its structural formula and molecular weight (Figure 1A). CCK‐8 experimental data stated that KPF was toxic to GM12878 cells at a dose greater than or equal to 240 μM (Figure 1B). Next, we further appraised KPF's mediation on DLBCL cell (OCI‐LY1 and U2932) proliferation. As illustrated in Figure 1C, OCI‐LY1 cell proliferation was repressed when KPF was greater than or equal to 15 μM, and the IC_50_ was 38.41 μM. Nevertheless, U2932 cell proliferation was suppressed when KPF was greater than or equal to 7.5 μM, and IC_50_ was 32.79 μM (Figure 1C). Hence, we chose KPF (7.5, 15, 30 μM) for the following analysis. Flow cytometry further proved that DLBCL cell apoptosis levels were enhanced with raised KPF doses (Figure 1D‐,E), but this impact was abolished after Fer‐1 treatment (Figure S1E). Collectively, these results indicated that KPF effectively suppressed DLBCL cell proliferation and induced apoptosis.

### 3.2 KPF Induces DLBCL Cell Ferroptosis

**FIGURE 2:**
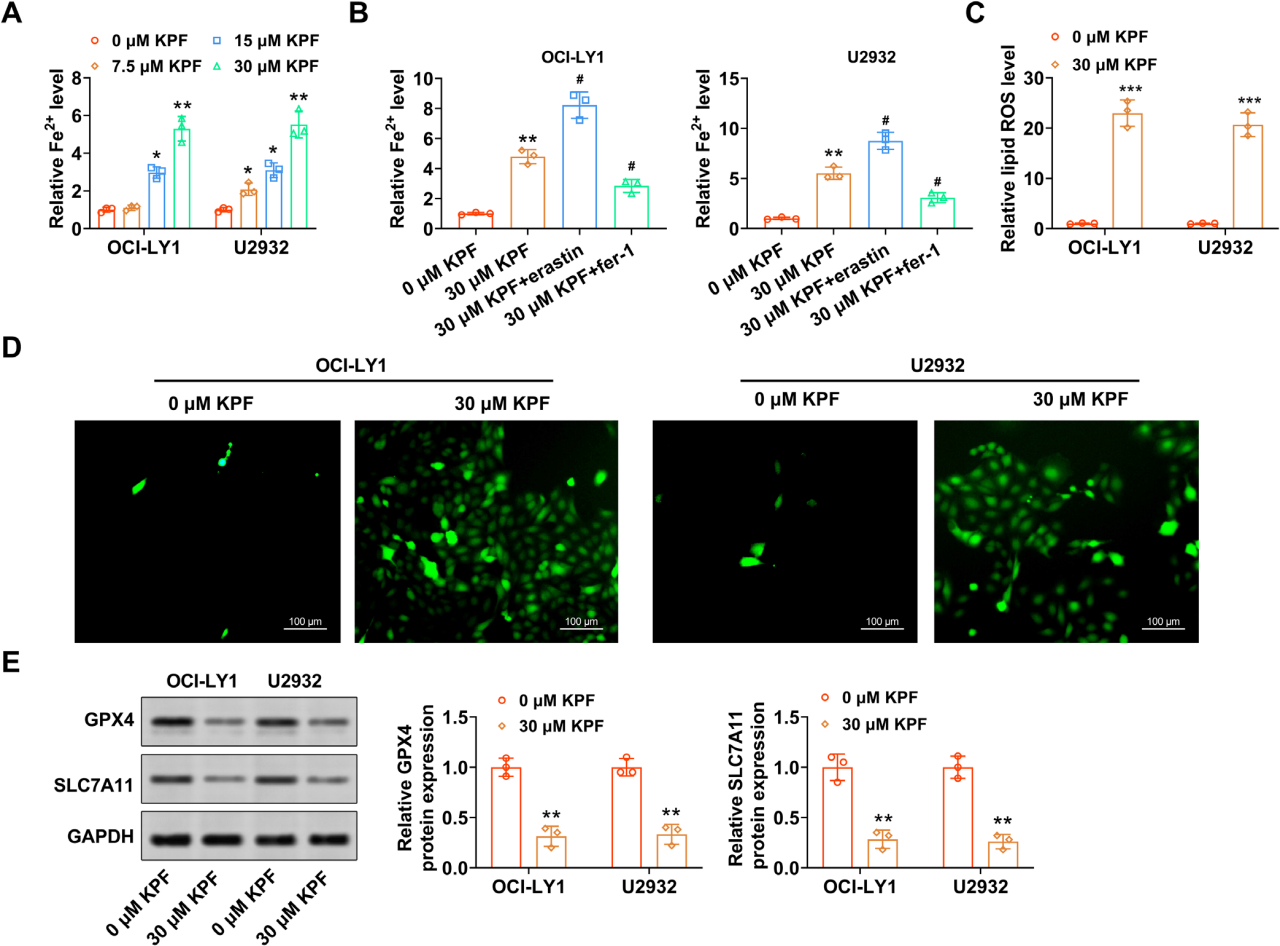
KPF promotes DLBCL cell ferroptosis. (A) DLBCL cells (OCI‐LY1 and U2932) were exposed to KPF (0, 7.5, 15, 30 μM) for 1 day. Contrast of Fe^2+^ levels in DLBCL cells using commercial kits. (B) After DLBCL cells were exposed to 30 μM KPF for 1 day, DLBCL cells were further exposed to 5 mmol/L erastin or 1 mmol/L ferrostatin‐1 for 1 day. Fe^2+^ levels in DLBCL cells were tested via commercial kits. (C, D) Reactive oxygen species (ROS) levels were checked using immunofluorescence (scale bar: 100 μM). (E) Western blot analysis of GPX4 and SLC7A11 (Ferroptosis signal axis‐related proteins) protein levels. *p < 0.05, **p < 0.01, ***p < 0.001 versus 0 μM KPF. ^#^ *p* < 0.05 versus 30 μM KPF.

Ferroptosis is an iron‐dependent form of regulated cell death characterized by the accumulation of lipid peroxides and oxidative damage to cellular membranes, and it has emerged as a promising therapeutic target in oncology. The underlying mechanism of ferroptosis is related to iron metabolism, lipid metabolism, and ROS accumulation. To investigate whether KPF induced ferroptosis in DLBCL cells, we treated OCI‐LY1 and U2932 cells with varying concentrations of KPF. As exhibited in Figure 2A, KPF reduced OCI‐LY1 and U2932 cell viability, while the apoptosis inhibitor benzyloxycarbonyl‐Val‐Ala‐Asp(OMe)‐fluoromethylketone (Z‐VAD‐FMK) and the ferroptosis inhibitor ferrostatin‐1 (Fer‐1) reversed this reduction (Figure S1A). Also, KPF raised Fe^2+^ levels in DLBCL cells, yet this effect was enhanced after erastin (ferroptosis activator) treatment, and ferrostatin‐1 (ferroptosis inhibitor) or NAC (a potent scavenger of ROS) abolished this impact (Figure 2B, Figure S1C,F). Meanwhile, KPF (30 μM) elevated ROS levels in DLBCL cells (Figure 2C,D), yet Fer‐1 or NAC abolished this trend (Figure S1D,G). Western blot further promulgated that KPF lessened GPX4 and SLC7A11 (ferroptosis signal axis‐related proteins) protein levels in DLBCL cells (Figure 2E), while this impact was abolished after Fer‐1 treatment (Figure S1B). These findings collectively demonstrated that KPF promoted ferroptosis in DLBCL cells.

### 3.3 KPF Inactivates NF‐κB Axis in DLBCL Cells

**FIGURE 3:**
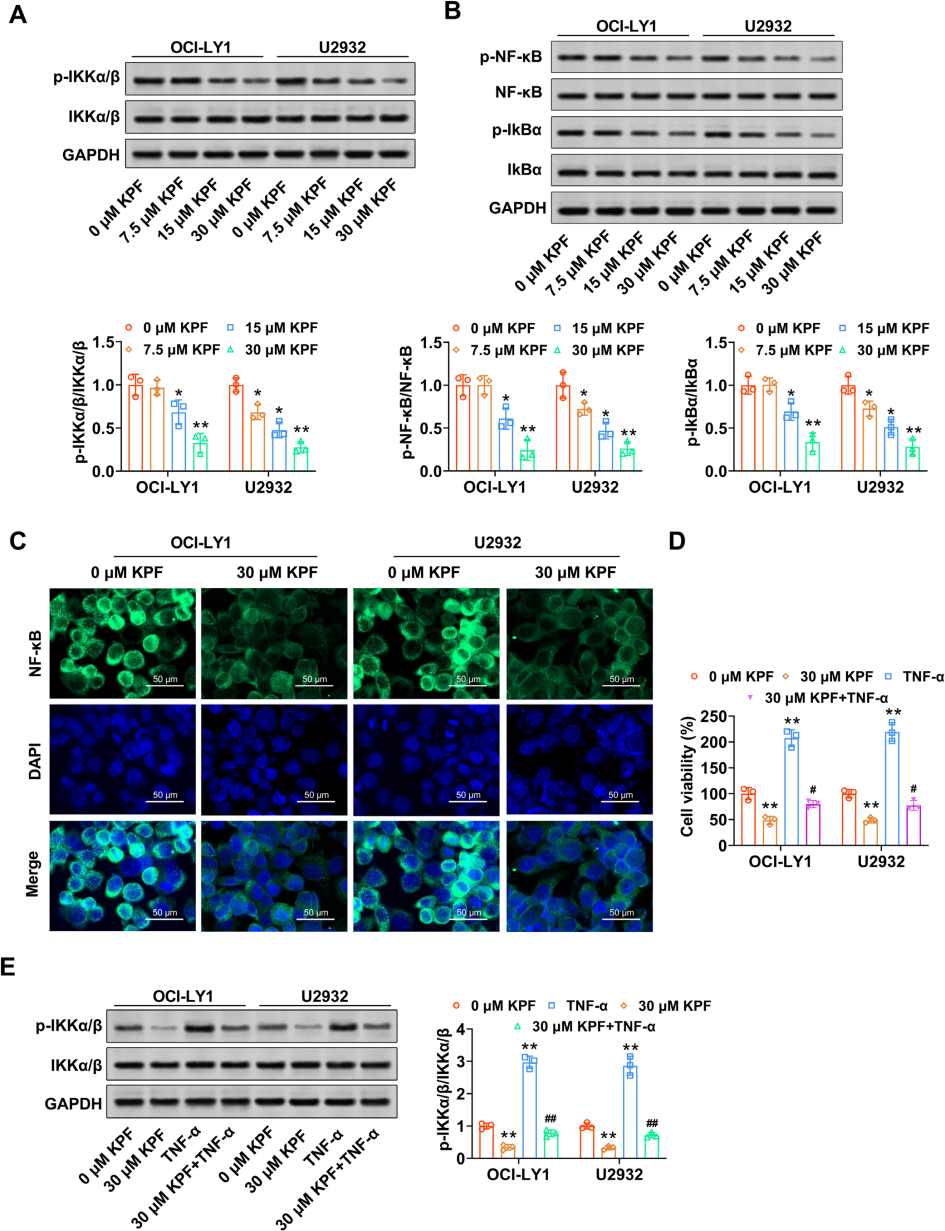
KPF represses the NF‐κB pathway in DLBCL cells. (A, B) DLBCL cells (OCI‐LY1 and U2932) were treated with KPF (0, 7.5, 15, 30 μM) for 1 day. P‐IKK α/β, IKK α/β, p‐NF‐κB, NF‐κB, p‐IKB‐α, and IKB‐α protein levels were appraised via Western blot. (C) DLBCL cells (OCI‐LY1 and U2932) were exposed to 30 μM KPF for 1 day. Immunofluorescence analysis of the NF‐κB entry into DLBCL cell nucleus (scale bar: 50 μM). DLBCL cells (OCI‐LY1 and U2932) were treated with KPF (30 μM) for 1 day and/or cells were further exposed to 5 ng/mL TNF‐α for 3 days. (D) DLBCL cell proliferation was assessed with the CCK‐8 assay. (E) P‐IKK α/β and IKK α/β protein levels were tested using Western blot. *p < 0.05, **p < 0.01 versus 0 μM KPF. ^#^ *p* < 0.05 versus 30 μM KPF.

To explore the potential molecular mechanisms underlying the anti‐tumor effects of KPF in DLBCL cells, we next evaluated its impact on the NF‐κB signaling pathway. As shown in Figure 3A, KPF (15, 30 μM) decreased the p‐IKK α/β (IKKα and IKKβ synergistically activate NF‐κB) protein levels in OCI‐LY1 cells, and KPF (7.5, 15, 30 μM) reduced the p‐IKK α/β protein levels in U2932 cells (Figure 3A). Western blot data promulgated that p‐NF‐κB and p‐IKB‐α protein levels were decreased in KPF (15, 30 μM) in OCI‐LY1 cells, and p‐NF‐κB and p‐IKB‐α protein levels were decreased in KPF (7.5, 15, 30 μM) in U2932 cells, yet IKB‐α and NF‐κB protein levels were unchanged between groups (Figure 3B). Moreover, KPF reduces NF‐κB entry into the DLBCL cell nucleus (Figure 3C). Meanwhile, 30 μM KPF reduced OCI‐LY1 and U2932 cell viability, while this impact was abolished after TNF‐α treatment. In addition, TNF‐α alone increased OCI‐LY1 and U2932 cell viability (Figure 3D). Also, 30 μM KPF decreased the protein levels of p‐IKK α/β in DLBCL cells, while TNF‐α treatment reversed this effect. Meanwhile, TNF‐α alone treatment increased the protein levels of p‐IKK α/β in DLBCL cells (Figure 3E). In summary, KPF restrained NF‐κB activation in DLBCL cells.

### 3.4 KPF Induces Apoptosis and Ferroptosis in DLBCL Cells by Inhibiting NF‐κB

**FIGURE 4:**
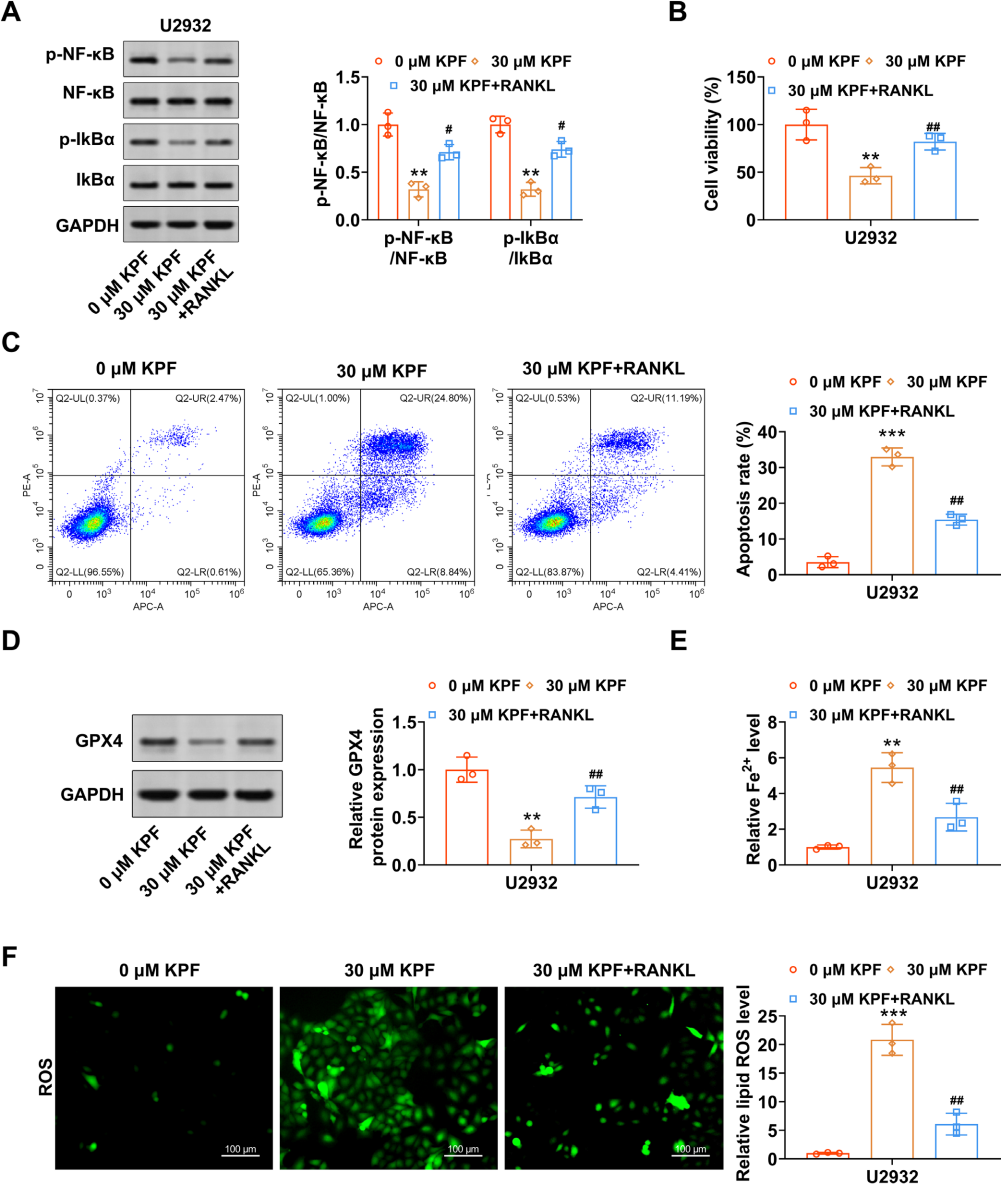
KPF induces apoptosis and ferroptosis in DLBCL cells by inhibiting NF‐κB. Followed by U2932 cells were exposed to 30 μM KPF for 1 day, and U2932 cells were then treated with 100 ng/mL RANKL (activator for NF‐κB) for 1 day. (A) Western blot analysis of p‐NF‐κB, NF‐κB, p‐IKB‐α, and IKB‐α protein levels. (B) U2932 cell proliferation was appraised using CCK‐8 assay. (C) Flow cytometry of U2932 cell apoptosis. (D) GPX4 protein levels were tested via Western blot. (E) Fe^2+^ levels were examined with commercial kits. (F) Immunofluorescence detection of ROS levels (scale bar: 100 μM). **p < 0.01, ***p < 0.001 versus 0 μM KPF. ^#^ *p* < 0.05, ^##^ *p* < 0.01 versus. 30 μM KPF.

Subsequently, we investigated whether the inhibitory effect of KPF on the malignant phenotype of DLBCL cells (U2932) was dependent on NF‐κB signaling. As shown in Figure 4A, KPF lessened p‐NF‐κB and p‐IKB‐α protein levels in DLBCL cells, and RANKL (activator for NF‐κB) counteracted this impact. Also, KPF weakened DLBCL cell proliferation capacity, yet this impact was abolished after RANKL co‐addition (Figure 4B). Flow cytometry presented the opposite trend in DLBCL cell apoptosis (Figure 4C). Our experimental data further elucidated that KPF reduced GPX4 protein levels in DLBCL cells, and RANKL counteracted this reduction (Figure 4D). Meanwhile, KPF raised Fe^2+^ levels in DLBCL cells, while RANKL counteracted the KPF elevation effect on Fe^2+^ levels (Figure 4E). Immunofluorescence analysis of ROS levels in U2932 cells showed a similar trend (Figure 4F). Collectively, these findings indicated that KPF suppressed the malignant phenotype of DLBCL cells through inhibition of the NF‐κB pathway.

### 3.5 KPF Represses DLBCL Cell Proliferation In Vivo

**FIGURE 5:**
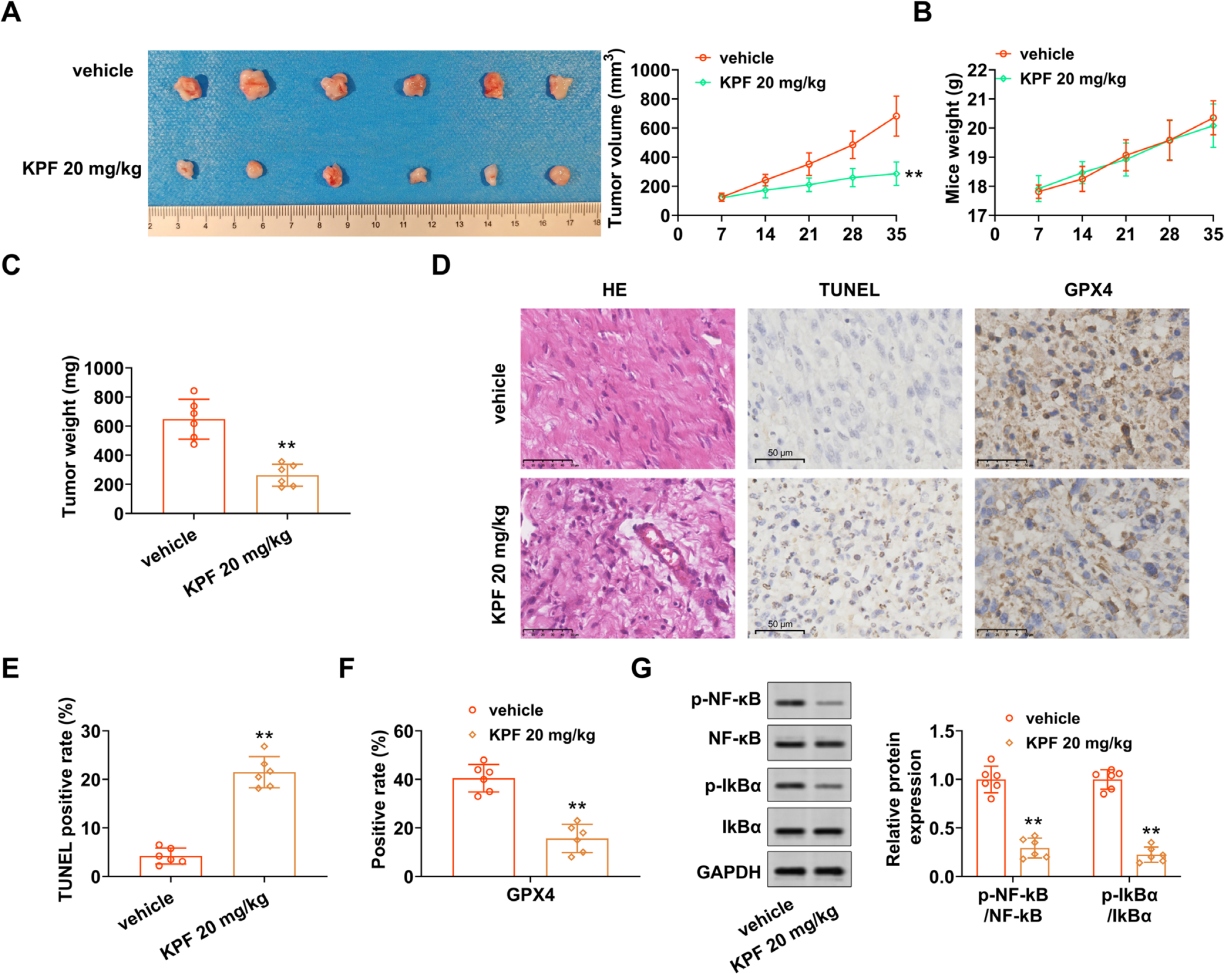
KPF reduces DLBCL growth in vivo. A mouse subcutaneous tumor model was constructed by injecting U2932 cells (1 × 10^7^) subcutaneously into mice, and 20 mg/kg KPF was intraperitoneally injected into mice. (A) Contrast of tumor volume. (B, C) Analysis of mouse weight and tumor weight. (D) Hematoxylin–eosin (HE) staining exhibited pathological changes of DLBCL (scale bar: 50 μM). (D, E) DLBCL apoptosis was examined using TUNEL (scale bar: 50 μM). (D, F) GPX4 expressions were appraised via immunohistochemistry (IHC, scale bar: 50 μM). (G) Comparison of p‐NF‐κB, NF‐κB, p‐IKB‐α, and IKB‐α protein levels with Western blot. **p < 0.01 versus Sham (vehicle).

Finally, we assessed the impact of KPF on DLBCL tumorigenesis using in vivo models. Here, we discovered that subcutaneous tumor volume was decreased after KPF treatment (Figure 5A). Moreover, KPF injection had no measurable effect on the mice's weight (Figure 5B), yet reduced the mice's tumor weight (Figure 5C). HE staining further proved that the disorder of DLBCL cancer tissue arrangement was alleviated after KPF treatment (Figure 5D). Besides, we expounded that DLBCL apoptosis in KPF was higher than that in sham (Figure 5D,E **‐**). IHC data stated that GPX4 expressions in DLBCL tissues were reduced after KPF injection (Figure 5D,F). Also, p‐NF‐κB and p‐IKB‐α protein levels were decreased in KPF (Figure 5G). To conclude, KPF reduced DLBCL growth in vivo. Overall, KPF promoted apoptosis and ferroptosis in DLBCL cells by suppressing the NF‐κB signaling pathway.

## 4 Discussion

DLBCL is the most common subtype of lymphoma, and there is currently a lack of effective therapy strategies for DLBCL [22]. In this study, we explored the role and potential mechanisms of KPF in DLBCL. Our study first demonstrated that KPF weakened DLBCL cell proliferation and enhanced cell apoptosis and ferroptosis. Moreover, we further elucidated that KPF restrained the DLBCL cell malignant phenotype via inactivating NF‐κB. Remarkably, KPF showed promising therapeutic effects in xenografted mouse models of DLBCL. To the best of our knowledge, this study provided the first direct evidence that KPF suppressed DLBCL progression through NF‐κB inhibition.

Flavonoids exhibit a wide range of biological activities, including antioxidant, anti‐inflammatory, neuroprotective, and anti‐aging properties [23]. In recent years, increasing studies have revealed the anti‐tumor function of flavonoids in diverse cancers, including DLBCL. Such as, Wogonin is an active flavonoid extracted from the traditional Chinese herb Scutellaria baicalensis, and represses DLBCL progression through PI3K and MAPK without significant side effects [24]. As a natural flavonoid, Luteolin induces apoptosis and cell cycle arrest in DLBCL cells, implying that Luteolin might be a potential therapeutic agent for DLBCL [25]. KPF is also named kaempferol‐3, 7‐rhamnoside, and KPF has protective effects against various human cancers. For instance, KPF induces apoptosis in hepatocellular carcinoma cells via the p21/Bcl‐2/Caspase‐3 pathway, providing a theoretical basis for its application in liver cancer therapy [10]. Additionally, KPF induces G1 phase cell cycle arrest in cervical cancer cells through a Caspase‐dependent intrinsic pathway, thereby exerting anti‐tumor effects [11]. Based on these findings, we sought to elucidate KPF's possible role in DLBCL. Remarkably, our experimental data revealed that KPF repressed DLBCL cell proliferation.

Apoptosis is a highly regulated form of programmed cell death, and the dysregulation and avoidance of apoptosis are cancer signs [26]. Most anti‐cancer therapies are designed to induce apoptosis in tumor cells, aiming to eradicate malignant cells [27]. Remarkably, KPF induces apoptosis in human malignant tumors, including liver cancer [10] and cervical cancer [11]. In the current study, our flow cytometry data highlighted that KPF promoted DLBCL cell apoptosis in vitro. Meanwhile, TUENL data further revealed that KPF induced apoptosis in DLBCL in vivo. These findings further supported the idea that KPF might enhance apoptosis in DLBCL.

Ferroptosis is a recently identified form of regulated necrotic cell death characterized by iron‐dependent lipid peroxidation, and ferroptosis‐induced PUMA plays an important role in the crosstalk between ferroptosis and apoptosis, suggesting that ferroptosis has a common pathway with other types of cell death [28, 29]. Ferroptosis is usually accompanied by a large amount of iron accumulation and lipid peroxidation [30]. Biochemically, intracellular glutathione depletion, glutathione peroxidase 4 (GPX4) activity decreases, and Fe^2+^ oxidizes lipids in a Fenton‐like manner to produce a large number of ROS, all of which can boost the ferroptosis occurrence [31]. In recent years, studies have proven that ferroptosis functions in the course of human diseases, including DLBCL, and has become a focus and hot spot in the study of treatment and prognosis improvement of DLBCL. For example, BRD4 inhibition sensitizes DLBCL cells to ferroptosis, supplying the rationale for novel therapies for DLBCL [32]. Dimethyl fumarate promotes DLBCL cell ferroptosis by inducing lipid peroxidation, hinting that dimethyl fumarate is a promising new therapeutic option for DLBCL [33]. As expected, our experimental data also state that KPF increased Fe^2+^ and ROS levels in DLBCL cells, while decreasing GPX4 and SLC7A11 protein levels in DLBCL cells. These studies further suggested that KPF had the potential to mitigate DLBCL.

Next, we focused on exploring the potential mechanisms by which KPF exerted anti‐tumor effects in DLBCL. Nuclear factor κB (NF‐κB) is a major transcription factor involved in immune‐mediated inflammatory response regulation [34]. The NF‐κB axis exists in numerous cells and is concerned with the regulation of various physiological and pathological processes, including cell proliferation, cell apoptosis, and ferroptosis [35]. Accumulating evidence suggests that NF‐κB dysregulation occurs in DLBCL processes. Zhong et al. indicated that fatty acid synthase restrains DLBCL cell ferroptosis by the activation of NF‐κB, suggesting a decisive role in mediating adriamycin resistance to DLBCL [36]. Guo et al. validated that miR‐525‐5p inhibits DLBCL progression through NF‐κB and provides a new reference for DLBCL‐targeted therapy [37]. Previous studies expound that KPF alleviates LPS‐induced acute lung injury in mice and reduces inflammation and apoptosis by NF‐κB [21]. KPF weakens cell proliferation, induces apoptosis, and improves inflammation of rheumatoid arthritis‐fibroblast‐like synoviocytes via inactivating NF‐κB [38]. Similarly, our data hinted that KPF reduced DLBCL malignant growth, whereas RANKL (activator for NF‐κB) abolished these impacts. Meanwhile, IkBα phosphorylation triggers the degradation of IκBα, but cells continuously synthesize new IκBα proteins through the NF‐κB‐dependent negative feedback cycle. Therefore, the total IκBα protein levels remain stable in the dynamic equilibrium between degradation and resynthesis [39, 40]. Remarkably, KPF also repressed DLBCL growth in vivo.

In summary, our study demonstrated that KPF induced DLBCL cell apoptosis and ferroptosis via inactivating NF‐κB. This might lay the experimental foundation for KPF development as a novel treatment strategy for DLBCL. Meanwhile, the limitations of this study were as follows: (1) Other relevant signaling pathways (such as PI3K/AKT/mTOR) have not been explored. (2) KPF roles in DLBCL malignant phenotypes with migration or epithelial‐to‐mesenchymal transition have not been probed (3) This study lacks pharmacokinetic or toxicity data to assess whether a relatively high concentration of KPF (240 μM) can be achieved and is safe for humans. We further explore and evaluate the drugability and safety of KPF in clinical use to further refine the conclusions of this study.

## Conflicts of Interest

The authors declare no conflicts of interest.

## Supporting information


**Figure S1:** (A) After DLBCL cells (OCI‐LY1 and U2932) were exposed to KPF (30 μM) for 1 day, cells were further exposed to 10 μM benzyloxycarbonyl‐Val‐Ala‐Asp(OMe)‐fluoromethylketone (Z‐VAD‐FMK, the apoptosis inhibitor) or 1 mmol/L ferrostatin‐1 for 1 day. Analysis of DLBCL cell proliferation using CCK‐8 assay. DLBCL cells (U2932) were exposed to KPF (30 μM) for 1 day, cells were further treated with 1 mmol/L ferrostatin‐1 for 1 day. (B) Western blot analysis of GPX4 and SLC7A11 protein levels. (C) Fe^2+^ levels in U2932 cells were tested via commercial kits. (D) ROS levels were assessed with immunofluorescence (scale bar: 100 μM). (E) DLBCL cells were exposed to 1 mmol/L ferrostatin‐1, and/or KPF (30 μM) for 1 day. DLBCL cell apoptosis was assessed by flow cytometry. DLBCL cells were treated with 30 μM KPF, 0.5 mmol/L *N*‐acetylcysteine (NAC, a potent scavenger of ROS), or 30 μM KPF combined with 0.5 mmol/L NAC for 1 day. (F) Fe^2+^ levels in DLBCL cells were measured with commercial kits. (G) Immunofluorescence analysis of ROS levels (scale bar: 100 μM). *p < 0.05 versus Fer‐1. **p < 0.01 versus 0 μM KPF or NAC. ***p < 0.001 versus 0 μM KPF. ^##^ *p* < 0.01 versus 30 μM KPF.

## Data Availability

The data that support the findings of this study are available from the corresponding author upon reasonable request.
