# Supplementary figures and images for: Kaempferitrin's Dual Assault: Inducing Apoptosis and Ferroptosis in Diffuse Large B‐Cell Lymphoma via NF‐κB Inactivation
Source: Kaohsiung J Med Sci. 2025 Oct 1;42(3):e70110. doi: 10.1002/kjm2.70110 (PMC12955990; doi:10.1002/kjm2.70110)

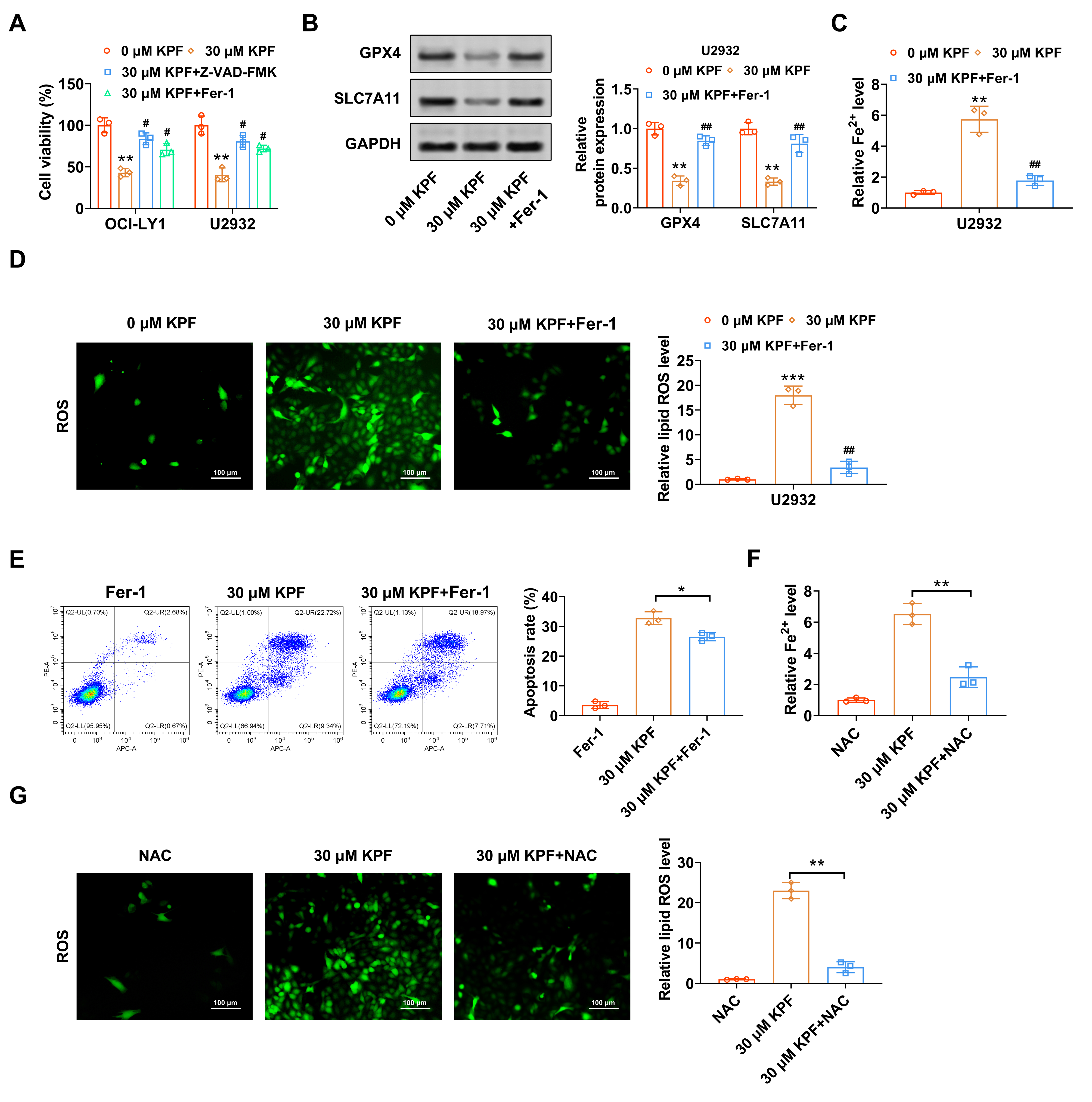

Supplement: Supplementary file 1 — Figure S1: (A) After DLBCL cells (OCI‐LY1 and U2932) were exposed to KPF (30 μM) for 1 day, cells were further exposed to 10 μM benzyloxycarbonyl‐Val‐Ala‐Asp(OMe)‐fluoromethylketone (Z‐VAD‐FMK, the apoptosis inhibitor) or 1 mmol/L ferrostatin‐1 for 1 day. Analysis of DLBCL cell proliferation using CCK‐8 assay. DLBCL cells (U2932) were exposed to KPF (30 μM) for 1 day, cells were further treated with 1 mmol/L ferrostatin‐1 for 1 day. (B) Western blot analysis of GPX4 and SLC7A11 protein levels. (C) Fe2+ levels in U2932 cells were tested via commercial kits. (D) ROS levels were assessed with immunofluorescence (scale bar: 100 μM). (E) DLBCL cells were exposed to 1 mmol/L ferrostatin‐1, and/or KPF (30 μM) for 1 day. DLBCL cell apoptosis was assessed by flow cytometry. DLBCL cells were treated with 30 μM KPF, 0.5 mmol/L N‐acetylcysteine (NAC, a potent scavenger of ROS), or 30 μM KPF combined with 0.5 mmol/L NAC for 1 day. (F) Fe2+ levels in DLBCL cells were measured with commercial kits. (G) Immunofluorescence analysis of ROS levels (scale bar: 100 μM). *p < 0.05 versus Fer‐1. **p < 0.01 versus 0 μM KPF or NAC. ***p < 0.001 versus 0 μM KPF. ## p < 0.01 versus 30 μM KPF. [file KJM2-42-e70110-s001.tif]
